# Development of a Detection Algorithm for Use with Reflectance-Based, Real-Time Chemical Sensing

**DOI:** 10.3390/s16111927

**Published:** 2016-11-16

**Authors:** Anthony P. Malanoski, Brandy J. Johnson, Jeffrey S. Erickson, David A. Stenger

**Affiliations:** Center for Bio/Molecular Science & Engineering, Naval Research Laboratory, Washington, DC 20375, USA; anthony.malanoski@nrl.navy.mil (A.P.M.); jeffrey.erickson@nrl.navy.mil (J.S.E.); david.stenger@nrl.navy.mil (D.A.S.)

**Keywords:** colorimetric, sensor, chemical detector, algorithm, autonomous

## Abstract

Here, we describe our efforts focused on development of an algorithm for identification of detection events in a real-time sensing application relying on reporting of color values using commercially available color sensing chips. The effort focuses on the identification of event occurrence, rather than target identification, and utilizes approaches suitable to onboard device incorporation to facilitate portable and autonomous use. The described algorithm first excludes electronic noise generated by the sensor system and determines response thresholds. This automatic adjustment provides the potential for use with device variations as well as accommodating differing indicator behaviors. Multiple signal channels (RGB) as well as multiple indicator array elements are combined for reporting of an event with a minimum of false responses. While the method reported was developed for use with paper-supported porphyrin and metalloporphyrin indicators, it should be equally applicable to other colorimetric indicators. Depending on device configurations, receiver operating characteristic (ROC) sensitivities of 1 could be obtained with specificities of 0.87 (threshold 160 ppb, ethanol).

## 1. Introduction

Recent effort has been focused on development of small, highly portable, chemical sensors that would address gaps in currently available technologies, allowing for autonomous and long term monitoring of contaminants in environmental air samples. The focus is on design of inexpensive devices with low power requirements. The approach is intended to facilitate the use of sensor arrays rather than single point detection units, providing information over a region of interest. Theoretical work has illustrated the potential benefits inherent in using chemical sensor arrays, rather than single devices, for obtaining early warning of threats as well as in gathering information on target distributions and plume movement [1]. The approach employed here uses semi-specific indicators that are differentially responsive across classes of targets rather than using specific sensors or indicators for each target [2]. The color changes in porphyrin and metalloporphyrin indicators upon interaction with targets provide the basis of the detection approach. The use of response profiles across multiple indicators offers the potential for a unique signature or “fingerprint”, providing resolution of targets to a class of chemicals and/or to a specific identification depending on the particular array of indicators used. Tracking of reflectance based color changes can be accomplished using low cost, commercially available sensor chips. We have previously reported on indicator behavior and initial prototype devices [2,3].

In addition to current hardware, software, and firmware components, a complete system requires development of an algorithm for identifying event occurrence and interpreting indicator responses. Our sensing approach is similar to that described by Suslick et al. [4,5,6,7] and others [5,6,8,9,10,11,12] in the use of reflectance based color changes; however, the particular array materials are different, having been selected for real-time detection applications, a consideration that also leads to strikingly different uses of the collected data. Previous work in this area focused on image processing to facilitate analysis of color changes and automation of this task as well as on characterizing the results for target identification [4,6,13,14,15,16]. There has been additional image analysis work specifically associated with the development of algorithms for use of colorimetric sensing with smartphones [17,18,19]. Unfortunately, most of this work is not applicable to the real-time detection approach taken under the current effort. Those efforts typically sampled a single time point following a specified exposure duration, rather evaluating a continuous data stream. In addition, the RGB (red, green, blue) color values reported were obtained from images (photographs) of the indicator arrays. RGB values for the current effort are directly reported by commercially available color sensing chips that are utilized in the prototype devices; images are not collected [2].

The algorithm described here focuses on identification of event occurrence. The larger effort seeks to develop an algorithm for identification of a detection event based on real-time or near real-time reporting of data in a continuous monitoring application. The algorithm will be tiered, first capturing all significant changes in color value so that only data from potentially significant events need be processed by higher tiers of the algorithm. The second tier of the algorithm would be used to identify whether detection events are related to compounds in a target library. This tiered algorithm structure evolved from potential operational strategies considered for reducing system costs through minimal onboard computations. Onboard software would address identification of an event, while higher tier analysis would be handled by a centralized processor receiving limited data streams from multiple individual detection devices. Other aspects of higher tier analysis could be developed to relate the spatial location of detection events using computational fluid dynamics [1]. This would allow for identification of other at risk locations and/or possible source locations. Here, we describe initial work based on the use of changes in standard deviation of color value data and the factors leading to use of a slope based approach for identification of events. While the algorithm developed will not provide a complete solution to real-time detection, the work represents an important step in advancing the utility of the sensing approach.

## 2. Materials and Methods

### 2.1. Sensor Systems

Two different device prototypes were used in the work presented, each of which controlled and collected data from six different commercially produced RGB color-to-frequency breakout boards (model TCS3200-DB, AMS-TAOS USA Inc., Plano, TX, USA). These boards provide a nonlinear response to changes in color intensity as well as overlapping spectral regions for the reported red, green, and blue color values. Technical specifications are provided by the manufacture. Each platform consisted of a custom printed circuit board (PCB) to control the hardware, timing, data collection, and to regulate and distribute power. Other components include sample holders, software interfaces, and in the case of the PT5, a housing with fans to provide airflow over the samples. PT3 is an early version of the prototype under development (Supplementary Materials, Figure S1). It was primarily designed to provide a system for screening of multiple indicator materials simultaneously, and it has been described in previous publications [2,3]. Details for this prototype are provided in the Supplementary Materials.

PT5 is a third generation prototype that has an enclosure as well as autonomous capabilities allowing for long duration unattended and outdoor operation (Supplementary Materials, Figure S2). Like the PT3, the sensor communicates sequentially with six copies of the TCS3200-DB board. The stock version of this board includes a color sensor (TCS-3200, AMS), an adjustable lens, and two white LEDs. Peak sensitivities for the TCS-3200 sensor occur at 470 nm, 524 nm and 640 nm. Cool white LEDs, however, tend to have low emission in the red portion of the spectrum, a region of interest for porphyrin-based detection. In order to enhance instrument sensitivity, one of the white LEDs on each detector board was removed and replaced with a 636-nm red LED rated at 1800 mcd at 20 mA forward current (product #SSL-LX5093SIC, Lumex, Austin, TX, USA). The two LEDs were aligned to the center of a target placed 2.54 cm away from the detector board prior to installation in the instrument.

The PT5 platform is designed to run outdoors autonomously for multi-day deployments. The housing is machined from chemically resistant Delrin plastic (McMaster-Carr, Robbinsville, NJ, USA) and secured with 316-stainless steel screws. Black plastic was chosen to minimize stray reflections. Plastic tipped thumb screws seal the top of the instrument for easy access in the field. All of the holes in the housing (cord pass-through; sample airflow) are located on the bottom of the instrument to provide protection from precipitation. The printed circuit board (PCB) that controls the instrument, the six TCS3200-DB breakout boards, the sample holders, and the two 5 V fans (model #OD2510-05HB, Orion Fans, Dallas, TX, USA; 2.7 cfm airflow over the samples) are all housed by the enclosure. PT5 has an upgraded microcontroller and flash memory suitable for deployment durations of up to 14 days. When using a previous version of this prototype, we observed that the timing of long deployments could be off by up to ±10%. PT5 incorporates a real time clock with a ±20 ppm quartz crystal, improving the 7-day timing accuracy from ±16.8 h to ±12 s. The power and controls for the fans have been incorporated into the PCB; one 7.5 V power supply (or set of batteries) is needed to power the instrument. In addition, the fans can be turned on and off by the firmware. This could be important for maintaining battery life in future studies with long intervals between samples; the total power draw from the fans (each one is about 160 mA at 5 V) is nearly ten times that of the rest of the instrument combined. PT5 allows the user to control both the sampling interval and the RGB integration time in software. Sampling interval choices used in this manuscript were 5 s and 30 s. The standard 100 ms integration time was available for both sampling intervals. For additional sensitivity, 200 ms, 300 ms, 400 ms or 500 ms integration times were available with the 30 s interval. Pseudocode for the firmware controlling the instrument is provided in the Supplementary Materials. A PC equipped with custom software (LabWindows, National Instruments, Austin, TX, USA) is used to start and stop each experiment and to download data from the instrument; connection to the PC is not required during experiments. Data acquired in real time is stored on flash memory and downloaded to the laptop over a USB connection upon completion of the experiment. For this report, four separate PT5 instruments are used. Humidity and temperature were tracked using an Omega Portable Data Logger with USB interface (OM-EL-USB-1, OMEGA Engineering, Inc., Norwalk, CT, USA).

### 2.2. Indicators

*meso*-Tetra(4-aminophenyl) porphine (N_4_TPP; CAS 22112-84-1) was obtained from Frontier Scientific (Logan, UT, USA). Ethanol was obtained from Warner-Graham (200 proof; Baltimore, MD, USA). Silver chloride (CAS 7783-90-6) was obtained from Sigma-Aldrich (St. Louis, MO, USA). The metalloporphyrin construct, AgN_4_TPP, used for collection of all data reported here, was prepared by reflux as previously reported [2,3,20]. Paper supported porphyrin indicator was prepared using a dip and dry technique. For a 5 cm × 33 cm swatch, 0.4 mM porphyrin in water (total volume 6 mL) was used. The paper support (WypAll X60, Kimberly-Clark Professional, Roswell, GA, USA) was pulled through this solution and allowed to dry slightly before being pulled through the solution again. This was repeated until all porphyrin solution had been deposited (typically three cycles). Samples were then dried at 100 °C before storing in the dark in sealed plastic bags. This procedure has been described previously for preparation of the indicator materials [2,3]. 

Two types of exposure experiments were conducted to evaluate sensor performance. The first type was utilized with only the PT3 prototype [2,3]. Porphyrin indicators were first measured over empty Petri dishes (60 mm diameter). The indicator supports were then moved to a warmed dish (60 °C) containing ethanol providing maximum initial ethanol headspace concentrations of 8, 16, 40, 61 or 82 ppm. Indicator supports were returned to the empty dishes for a minimum of 5 min prior to initiation of another exposure event. This type of exposure provides a brief peak target concentration with ongoing air exchange; reported values are the highest possible concentration based on target volume used [2]. The second type of measurement provided a single headspace for all indicators and devices. Devices were used for continuous data collection over periods of up to fourteen days encompassing multiple exposure/purge periods. For this measurement, the sensors with mounted indicator materials (six AgN_4_TPP indicators per device) were placed in a glove chamber (65 L; Techni-Dome, Bel-Art, Wayne, NJ, USA), and the volume was purged with humidified air for establishment of baseline measurements. Exposures were accomplished by adding a fixed volume of target to the continuous air stream to produce maximum ethanol concentrations of 0.05, 0.16, 0.32, 0.53, 1.06 and 1.58 ppm. 

### 2.3. Scripts

The original sensor prototype (PT3) contained an infrequent reporting anomaly that lead to an intensity counter being prematurely dumped; the result is a very low (often zero) reported value for an individual color channel (R, G or B on a single sensor). These anomalous data values were excluded in the first round of processing by comparing the channel value to that reported for the previous time point. If the absolute value of the difference between the two divided by the previous value was greater than 35%, the previous value was substituted for the current value. This approach allows for application of the algorithm to live or stored data; all detection algorithms were designed to function in real-time or when applied to stored data. Though the color sensing chips report a white channel, this information is ignored by all algorithm variations.

#### 2.3.1. Standard Deviation Based Algorithm

Initial detection criteria are based on examination of the red, green, and blue color channels for each indicator. For analysis at a given time point, the standard deviation for each color channel (RGB) of a single indicator (seat) is computed using the 12 most recent time points. The RGB standard deviations are subsequently divided by the average intensities for the same 12 points. If the resulting value for all three color channels is greater than 0.00015 or if any two of the values are greater than 0.015, this is classified as a potential detection event. The device (six indicators) is considered to have detected an event of interest when at least the specified minimum number (one, two or three) of indicators (seats) report a detection event at the same time. After event detection, a time window of 30 min is activated. Any simultaneous indicator events that meet the specified minimum within this window are considered part of the initial event. The window length is extended by 30 min each time an event meeting the criteria is identified. Following 30 min without additional event detection, the time stamps for the first and last detected events are reported along with the identification of all indictors that met event detection requirements within the time period. 

**Figure 1:**
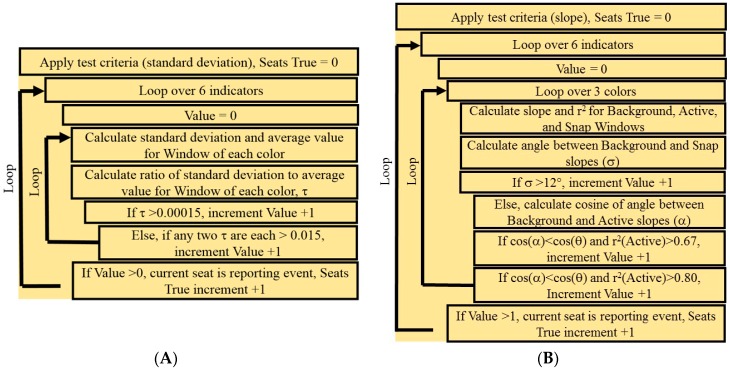
(**A**) Psuedocode describing the test criteria utilized for the standard deviation based algorithm (std); (**B**) Psuedocode describing the test criteria utilized for the slope based algorithm (s1, s2, s3).

A detailed pseudocode description for the full process is provided in the Supplementary Materials. Figure 1 provides a description of the standard deviation test criteria.

#### 2.3.2. Slope Based Algorithms

For application of the slope based algorithm, the threshold angle is fixed for each color value of each indicator based on the first 120 data points following initiation of the device. The initial intensity for each color is used in the following formula:

(1) $$\emptyset =ae^{\frac{-RGB}{b}}+c$$

RGB is the initial intensity for a color channel. The parameters used depend on the minimum number of indicators or on the initial intensity values with *a* = 20 (one indicator, 70 for more than one indicator), and *b* = 130 (one indicator, 30 for more than one indicator), and *c* equal to the larger value of a user specified value (default 0.45°) or the angle of the dot product of the standard error calculated for the first 120 data points following initiation of the prototype. 

Linear regression formulas are used to compute the slope and *r*^2^ value for each color channel over two windows, current and baseline. The current window comprises the 20 (30 s sampling interval; 10 min) or 50 (5 s sampling interval; 4.2 min) most recent time points; the baseline is a window that comprises the next 120 most recent time points. The cosine of the angle between the slopes for the baseline and current windows is computed. If this value is less than the cosine of the threshold angle (above) and the *r*^2^ value for the current window is greater than 0.67 (one indicator, 0.57 for more than one indicator), the color value is counted as 1; if the *r*^2^ value was greater than 0.8, the color is counted as 2. Counts from all color channels for a given indicator must sum to greater than 1 for the indicator to be considered to have detected a change. Very high dose exposures can induce large or rapid changes; to address this situation, an additional test was added that uses only the 10 most recent data points. If the angle between the computed slope of this window and the reference region slope is greater than 12° then the color contributes 1. After these tests have been applied to all indicators, cases in which the number of indicators that have detected changes is greater than or equal to the minimum number specified are considered to have detected an event. Similarly to the standard deviation algorithm, an event window was used for determination of the end point. Here, that window is 60 min rather than 30 min. Any indicator events within this window extended the event window by 60 min until no events are detected. The time stamps of the first and last detected events are reported along with indicator identification for all indicators reporting within the window. A detailed pseudocode description of the process is provided in the Supplementary Materials; the test criteria are outlined in Figure 1.

## 3. Results and Discussion

**Figure 2:**
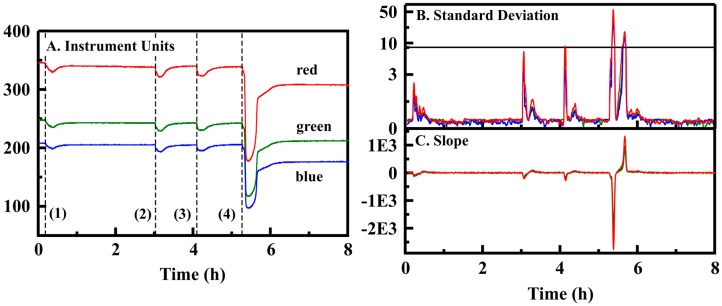
(**A**) PT3 data for a single indicator (RGB values, 5 s sampling interval) with multiple exposures (indicated by dashed lines) at maximum ethanol concentrations of 8 (1, 2 & 3) and 16 ppm (4); (**B**) Standard deviation over time calculated using a 12 point sliding window for presented data; (**C**) Slope over time calculated using a 50 point sliding window for presented data.

Initial development of a detection algorithm focused on the use of a standard deviation based approach to identification of an event. The work utilized data sets that have been reported on previously [2]. These were collected with the PT3 prototype applied in Petri dish evaluations for screening of the various porphyrin and metalloporphyrin indicators and included exposures to ethanol, methanol, isopropanol, acetone, acetic acid, and methyl salicylate [2]. Assessment and tuning of the developed algorithms was accomplished using data sets generated with a single indicator, silver *meso*-tetra(4-aminophenyl) porphine (AgN_4_TPP), with a single target, ethanol. For the PT3 prototype, 180 exposure events at five concentrations were completed in the Petri dish configuration. These evaluations included continuous data collection over periods of up to 8 days (Supplementary Materials, Tables S1 and S2). The results reflect those obtained under the original indicator screening effort [2]. Figure 2 presents representative data for Petri dish exposures of the PT3 prototype along with standard deviation calculated in the manner used by the algorithm. The algorithm uses changes in the standard deviation of two or more color values for a single indicator to provide identification of an event (s1std). This approach provided the potential for rapid detection (1 min or less) with minimal instrument cycle up time (1 min). In combination with the PT3 prototype and this type of evaluation, the sensitivity (or true positive rate) was 0.87; the specificity was 0.92 for a detection threshold of 8 ppm. Altering the algorithm to require changes on two or more seats (s2std) for event identification lead to a slight improvement in specificity (0.96) and sensitivity (0.93).

**Figure 3:**
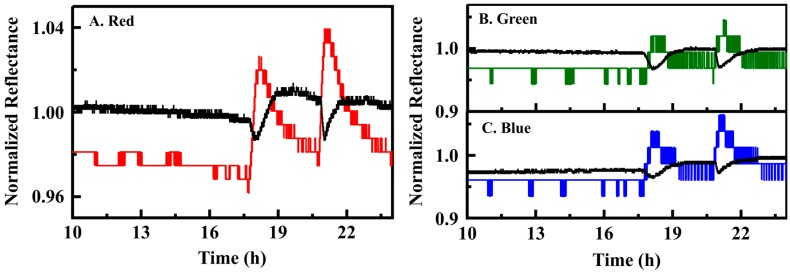
PT3 data at a 5 s sampling interval (100 ms integration; PT3; black) compared to 100 ms PT5 data (RGB, 5 s sampling interval). The two exposures shown here were completed in the glove enclosure using a maximum ethanol concentration of 1.06 ppm.

PT3 is a bench-scale prototype designed primarily for screening of indicator materials (Supplementary Materials, Figure S1). Petri dish based exposures were effective for identification of indicator array components, assessment of response times, and determination of clear down rates associated with array elements but have little relevance to the environmental sensor system under development. Exposure of the device to targets within an enclosed headspace was intended to provide a better simulation of the intended application conditions [2]. With this approach, target concentrations can be rapidly increased from baseline to the desired level. Return to baseline conditions occurs as the headspace is purged by air flow. Figure 3 provides representative data for the PT3 prototype under this type of evaluation. 

In this case, the presented data has been normalized to the average intensity value to facilitate comparisons across devices. The time dependence of the color values reflects the increase to peak concentration, slower than that observed in the Petri dish format, and the slow return to baseline conditions as the air within the enclosure is exchanged. This format allows for interrogation of ethanol concentrations between 50 ppb and 1.6 ppm; 138 exposures of PT3 at five concentrations (0.16 to 1.6 ppm) were completed in this format (Supplementary Materials, Table S3). When the standard deviation algorithm (s1std) was applied to the resulting data set, the sensitivity was 0.07; the specificity was 0.99 for a detection threshold of 160 ppb. Table 1 provides ROC analysis results for algorithms applied to PT3 data sets (see also Supplementary Materials, Table S4).

While the standard deviation based algorithm provided poor performance when applied to the PT3 data sets collected in the glove enclosure, manual analysis indicated that the sensor system was capable of providing ethanol detection at even the 160 ppb level. At this point, analysis of data collected using PT5 prototype devices began (four independent, six element devices). The PT5 devices were designed to function autonomously for periods of up to 14 days (30 s sampling increment) after which time the data was manually downloaded (Supplementary Materials, Figure S2). These devices were exposed to targets in parallel with the PT3 device using the glove enclosure procedure. A data set comprising 384 exposures at five concentrations between 0.16 and 1.6 ppm was collected using PT5 (100 ms integration, 5 s interval; Supplementary Materials, Table S5). Application of the single seat standard deviation based algorithm (s1std) to this data set resulted in a single reported event, a false positive. The dual seat version (s2std) reported no events (Supplementary Materials, Table S6). 

Based on these results, a second algorithm (s1; Section 2.3.2) was developed with detection based on changes in the slope of the color value data. This algorithm provided a sensitivity of 0.50 with specificity at 0.90 for the PT5 data set (Table 1). When applied to the PT3 data set, sensitivity was dramatically increased over the standard deviation based algorithm (0.82 vs. 0.07) with additional improvement in the specificity (0.998). Interestingly, application of the slope based algorithm to the data set collected using Petri dish exposures resulted in sensitivity 0.86 with specificity 0.95. The reasons for the variation in algorithm performance can be seen when the results reported in Figure 1 and Figure 2 are compared. One contributing factor was the differences in experimental procedures. The standard deviation based algorithm was more effective for the sharp concentration changes achieved in the Petri dish format, but was poorly suited to the slightly more gentle increase in concentration of the glove enclosure experiments. The performance of the two prototype devices leads to some variation as well. The average color value for PT3 is between 391 and 569 for the AgN_4_TPP indicator depending on the channel (RGB) analyzed (average 466); for PT5, it varies from 113 to 27 (average 56). The standard deviation in the PT3 data is less than 1% of the total signal; for PT5, it is up to 5% depending on the color channel evaluated. These variations are a result of manufacturing differences in the breakout boards used (see Methods). 

**Figure 4:**
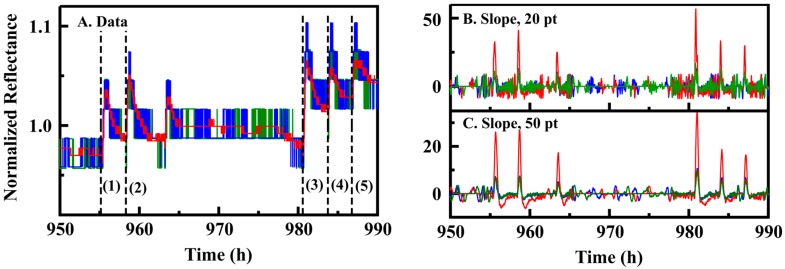
(**A**) PT5 data (PT5; 30 s sampling interval; 100 ms integration) is presented including points collected during exposure to ethanol in the glove enclosure at maximum concentrations of 1.06 ppm (1, 2) and 1.58 ppm (3, 4, 5); The reported signals are red, green, and blue values for a single indicator. Also shown is the calculated slope versus time for the presented data using varied window lengths of 20 (**B**); and 50 (**C**) points.

In order to achieve 14 days of autonomous data collection, the PT5 devices use a 30 s sampling increment; the 5 s sampling increment, used for comparison to the PT3 device, provides 60 h of autonomous data collection. It is desirable to keep the sampled window as short as possible in order to make real-time application of the algorithm possible. With this in mind, the algorithm was adjusted to use 20 points rather than the 50 utilized with the 5 s sampling increment keeping the total time at 10 min. The use of 20 points maintained high sensitivity while introducing few false positives (Figure 4). Application of this variation of the slope based algorithm (s1) yielded a specificity of 0.94 with a significant loss in sensitivity (0.23). Because the final versions of the sensor device will probably require multiple indicators for identification of an event, it was recognized that thresholds could be changed to potentially increase sensitivity with specificity being maintained by requiring responses from more than a single indicator. A two seat variation of this algorithm (s2) provided somewhat improved sensitivity 0.48 (0.98 specificity). Increasing the concentration limit from 0.16 to 1.58 ppm provided a sensitivity of 0.88 (for s2; s1 = 0.61). This sensitivity was still below that sought for the intended application, and the concentration threshold is much higher than that desired. 

Down sampling the PT3 data set to a 30 s interval did not cause these types of losses in sensitivity and specificity (Supplementary Materials, Table S4). PT3 reports significantly higher color values than PT5 (100 ms integration). This result lead to consideration of device performance. It is possible to increase the total signal for the PT5 device through increasing the integration time used; the standard deviation in the signal simultaneously drops. Increasing the integration to 200 ms, increased the average color value to 231 with a standard deviation of 2.8%. At 500 ms integration, the maximum possible at this sampling interval, the average color value was 713 with a standard deviation of 0.7% (Full summary provided in the Supplementary Materials, Table S7).

**Figure 5:**
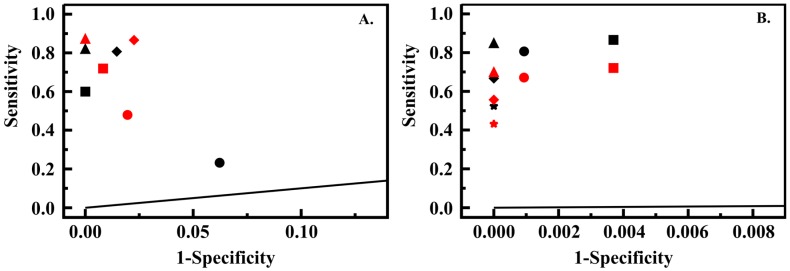
(**A**) ROC analysis for PT5 data sets collected at 100 (circle), 200 (square), 400 (triangle) and 500 ms (diamond) integration when evaluated using the single seat (s1, black) and dual seat (s2, red) slope based algorithms; (**B**) ROC analysis of the 500 ms PT5 data set using 0.16 (black) and 0.05 (red) ppm detection thresholds with variations of the slope based algorithm: one seat, 0.45°, circle; two seat, 0.45°, square; three seat, 0.45°, triangle; one seat, 1.45°, diamond; one seat, 2.45°, star.

To evaluate the impact of total signal and noise levels on algorithm performance, PT5 data sets were generated for 200, 400 and 500 ms integration times. These sets consisted of 360 exposures at five concentrations between 0.16 and 1.6 ppm. Figure 5 provides ROC analysis for the single (s1) and dual (s2) seat slope based algorithms across the four integration times utilized. Increasing integration to 200 ms dramatically improved both sensitivity and selectivity over that observed for the 100 ms data set. Additional improvement was obtained using 400 ms integration. Increasing integration time to 500 ms did not result in additional gains; in fact, a loss in specificity was noted under both variations of the algorithm. 

For further tuning of the algorithm, the 500 ms integration data set was expanded to include 72 exposures at a concentration of 0.05 ppm (Supplementary Materials, Table S8). This data set was analyzed using the single seat slope based algorithm at a detection threshold of 0.16 ppm as well as 0.05 ppm. The angle threshold for detection was varied at 0.45°, 1.45° and 2.45°. The dual seat slope based algorithm (0.45°) was applied along with an additional variant of this algorithm using a three seat requirement (0.45°). As shown in Figure 5, specificity could be increased at the expense of sensitivity through increasing the angle threshold value. The three seat variant of the algorithm using the lowest angle threshold provided the best performance overall, returning no false positives with a sensitivity of 0.85 (0.16 ppm threshold). The 0.05 ppm level exposures were not detected by any of the algorithm variants. If the concentration threshold is shifted to 0.32 ppm, the two seat and three seat variants identify all events with no false positives (sensitivity and specificity both equal 1).

## 4. Conclusions 

A reasonable algorithm for identification of a detection event in real-time sensing applications that employ reversible colorimetric indicators based on changes in the slope of collected data has been described. The method focuses on the identification of event occurrence, rather than target identification, and utilizes an approach suitable to incorporation onboard a device intended for portable, autonomous use. The described approach first excludes electronic noise generated by the sensor system. Multiple signal channels (RGB) as well as multiple array elements are combined for reporting on an event with a minimum of false responses. The method automatically adjusts to the overall intensity of the data collected, facilitating use with different device variations as well as accommodating differing indicator behaviors. While the method reported was developed for use with paper-supported porphyrin and metalloporphyrin indicators, it should be equally applicable to other colorimetric indicators. The approach is specifically suited to reversible indictors, but could be utilized with reactive indicators as well.

The algorithm development process allowed for identification of important physical device parameters prompting adjustments to the prototype. It is clear that the integration time and sampling frequency effect the limits of detection for the device leading to different versions of the device achieving similar sensitivities at differing integration times. The effect of integration time on the signal to noise ratio is readily apparent. Longer integration times, up to 400 ms, improved sensitivity for the PT5 device but little to no difference was seen from 400 to 500 ms. In the current device, use of 400 to 500 ms integration limits data collection to a 30 s sampling increment. There are indications that shorter sampling increments would result in improved performance at lower threshold target concentrations; however, this point could be investigated with only the PT3 device. 

It is important to note that, while the points used in the detection window cover 10 min (30 s sampling increment), the device consistently indicated the beginning of an event within 1 or 2 min following target introduction. The optimal sampling frequency will ultimately depend on limitations of the sensor materials and the desired application. For the current application, the 30 s sampling increment is preferred as data is stored in device memory for later download; a longer sampling increment increases the time between downloads. In order to more fully explore optimal operating conditions, device modifications allowing shorter sampling increments without losses in integration time are necessary. Further effort will consider these hardware upgrades as well as incorporating the described algorithm in device firmware as a first step toward automated reporting.

## Figures and Tables

**Table 1:** ROC comparison for algorithms applied to PT3 data collected using either Petri dish or glove enclosure exposures. Analysis of PT5 data collected at 100 ms with a 5 s sampling interval is also included.

Device	Experiment Type ^1^	Algorithm Type	Specificity	Sensitivity
PT3	Petri dish	s1std	0.92	0.87
PT3	Petri dish	s2std	0.96	0.93
PT3	Enclosure	s1std	0.99	0.07
PT3	Enclosure	s2std	0.99	0.07
PT5	Enclosure	s1std	1.00	0.00
PT3	Petri dish	s1	0.95	0.86
PT3	Enclosure	s1	1.00	0.82
PT5	Enclosure	s1	0.90	0.50

^1^ Petri dish based exposures utilized ethanol concentrations from 8 to 82 ppm; enclosure exposures were 0.16 to 1.58 ppm.

## Supplementary Materials

The following are available online at http://www.mdpi.com/1424-8220/16/11/1927/s1, Details on the Multiplex Development Platform (PT3), Pseudocode for PT3, Pseudocode for PT5, Figure S1: Photograph of PT3, Figure S2: Photographs of PT5, Pseudocode for the algorithms developed, Table S1: Data set from PT3 petri dish exposures—175 h, Table S2: Data set from PT3 petri dish exposures—334 h, Table S3: Data set from PT3 enclosure exposures—1004 h, Table S4: Results for analysis of PT3 data sets, Table S5: Data set from PT5 enclosure exposures at 100 ms integration—243 h, Table S6: Results for analysis of PT5 data sets, Table S7: Sensor performance at varied integration time, Table S8: Data set from PT5 enclosure exposures at 500 ms integration—510 h.
