# Supplementary material for: Development of a Detection Algorithm for Use with Reflectance-Based, Real-Time Chemical Sensing
Source: Sensors (Basel). 2016 Nov 16;16(11):1927. doi: 10.3390/s16111927 (PMC5134586; doi:10.3390/s16111927)
Supplement: Supplementary file 1 [file sensors-16-01927-s001.pdf]

Supplementary Materials: Development of a Detection Algorithm for Use with Reflectance-Based, Real-Time Chemical Sensing

Anthony P. Malanoski, Brandy J. Johnson, Jeffrey S. Erickson and David A. Stenger

Multiplex Development Platform (PT3)

Two different sensor platforms were used in this work, each of which controlled and collected data from six different commercially produced RGB color-to-frequency breakout boards (model TCS3200-DB, Rocklin, CA, USA). Each platform consisted of a custom printed circuit board (PCB) to control the hardware, timing, data collection, and to regulate and distribute power. Other components include sample holders, software interface, and in the case of the PT5, a housing with fans to provide airflow over the samples.

The first platform, labeled PT3, has been described previously (Johnson et al. (2014), doi:10.1088/0957-0233/25/9/095101). The PT3 uses flexible cables to connect six of the RGB sensors to a custom circuit board; the RGB sensors are mounted to in-house developed sample holders machined from chemically resistant Delrin plastic. The sample holders are designed to provide a working distance of one inch from sample to sensor using copper spring clips (Ted Pella, Redding, CA) to mount the indicators and are open at the bottom to allow vapor to interact with the targets (Figure S1). The Delrin bases are designed to serve as lids for 60 mm Petri dishes. Each RGB sensor has two white LEDs mounted at 45-degree angles to the indicator surface. When activated, each sensor pulses the LEDs for 800 ms, during which the red, green, blue, and clear channels are measured. Integration time for each channel is fixed at 100 ms. Each set of LEDs are on only during the 800 ms interval when the sample is being actively probed in order to minimize any photobleaching as well as power requirements. The six RGB sensors are sampled sequentially (rather than simultaneously) due to limitations in the TCS3200-DB breakout board design; here, one cycle of data collected in either a 5 s or a 30 s sampling interval. Regardless of the interval, the RGB sensors are activated 800 ms apart, with all six sensors collecting and reporting data 4.8 s into the cycle. On a 5 s sampling interval, the instrument pauses for 200 ms before starting the next cycle. For a 30 s cycle, the instrument pauses for 25.2 s.

RGB measurements collected by the TCS3200-DB breakout boards are converted to a sequence of digital pulses proportional to the intensity values and sent to the PT3 microcontroller. The PT3 board counts the pulses over each 100 ms integration period and stores the results in flash memory. In addition, the microcontroller controls sensor timing and distributes regulated power to the entire system from the USB supplied 5Vsource. Alternatively, the PT3 can be powered through its DC barrel jack connected to either batteries or a 7.5 V AC/DC adapter.

A custom graphical user interface (GUI) was developed using LabWindows software (National Instruments, Austin, TX, USA); this interface communicates with PT3 through simple ASCII commands such as start, stop, unload data stored in firmware, and perform a flash memory erase. The PCB firmware is designed to run continuously once started, stopping only if the flash memory becomes full, the stop command is received, or a manual reset is performed. The firmware sends out data after every measurement cycle, so that if a computer is connected the results can be viewed on the GUI as they are received. The firmware requires no acknowledgement or handshaking during regular operation; it will continue to run even if the computer is disconnected or otherwise not available to receive data.

PT3 Pseudocode

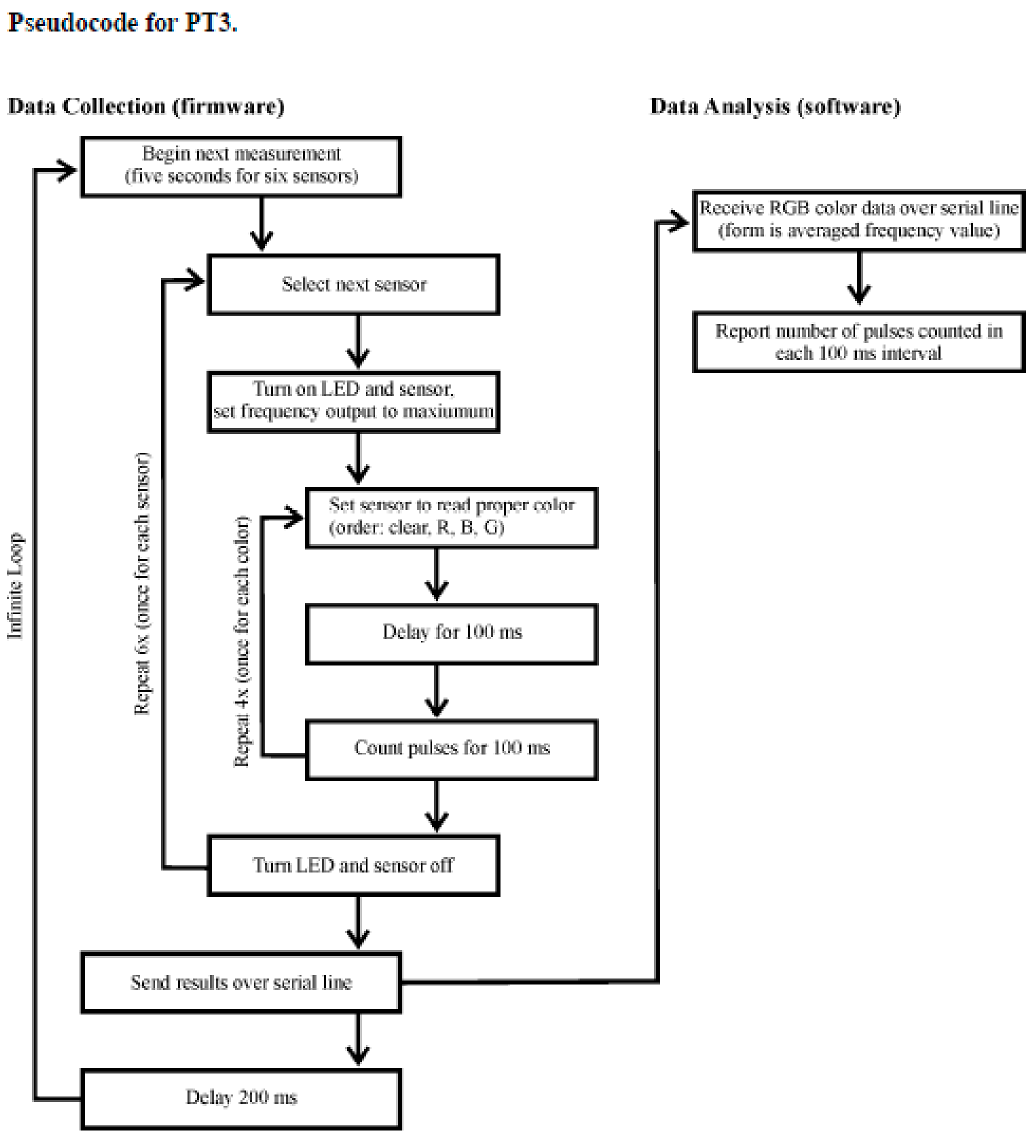

Prototype Sensor Device (PT5)

The most important firmware details for the operation of PT5 are two counter/timers and a status flag. The real time counter (RTC) controls all timing events for the instrument. The RTCs period (5 s or 30 s, corresponding to the sampling interval) and its capture/compare value (the integration time, 100–500 ms) are variables and are set at the beginning of an experiment. The pulse counter is a 16-bit counter/timer used to integrate the signal from each color channel. It is reset after each individual measurement. The status flag tells the firmware what the instrument is currently doing. It has the following values:

0—RTC overflow event has occurred/start new measurement cycle
1—instrument is waiting for the TCS3200 sensor to stabilize
2—instrument is counting pulses over an integration period
3—finished counting all sensor channels/instrument is waiting for the RTC to overflow
4—stop command received and/or TCS3200 sensors are powered off

PT5 Pseudocode

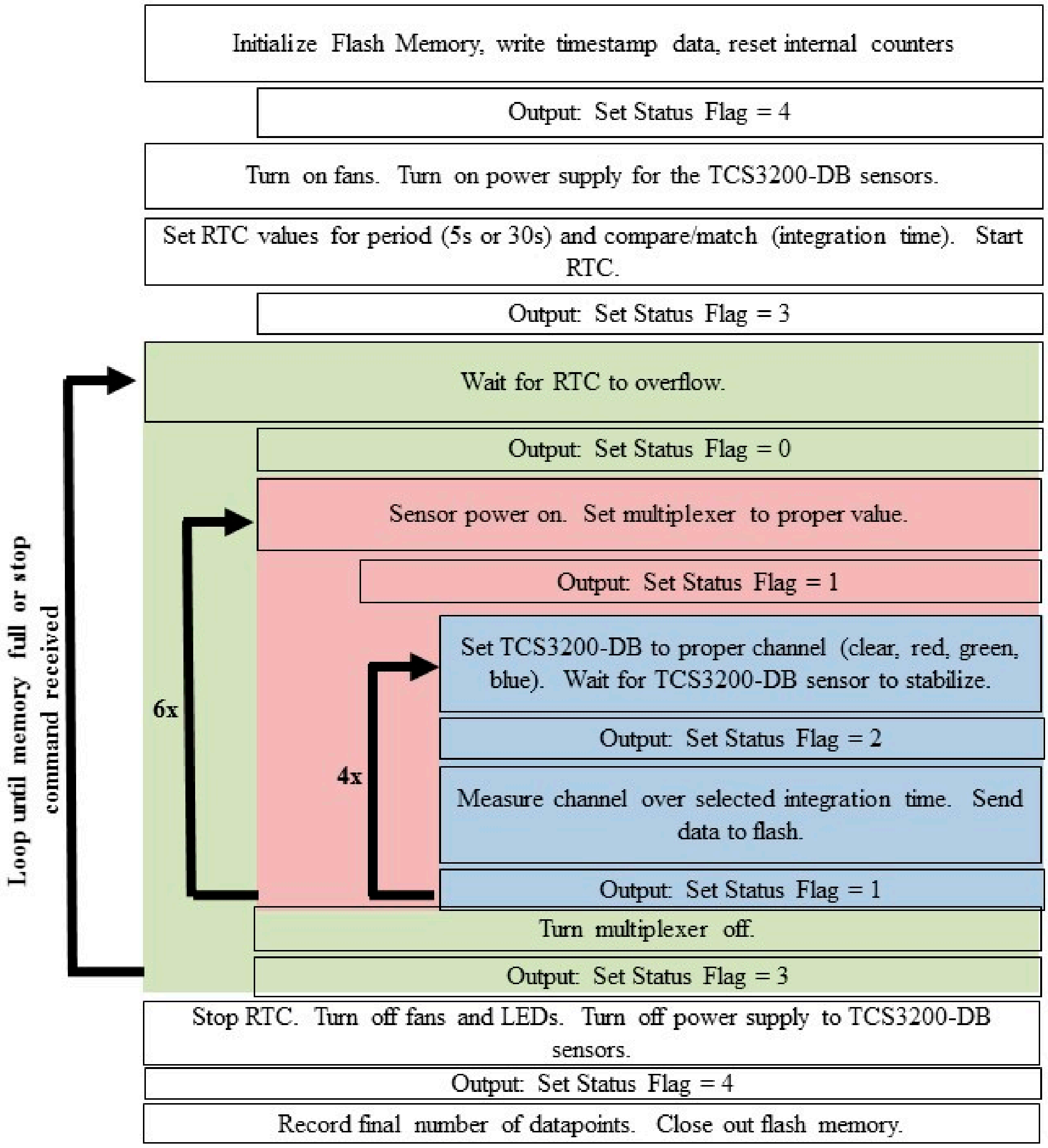

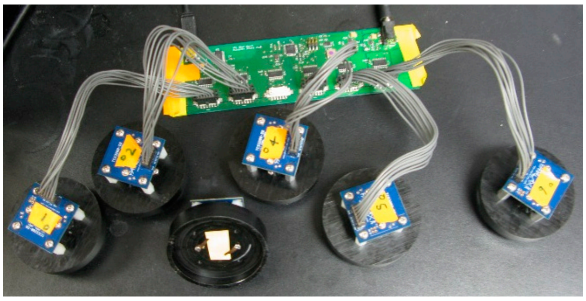

Figure S1. PT3 prototype device.

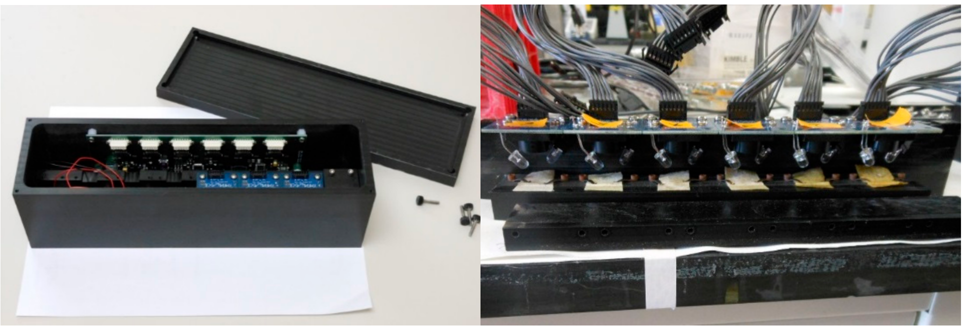

Figure S2. PT5 prototype device.

Pseudocode for Algorithms

Overview

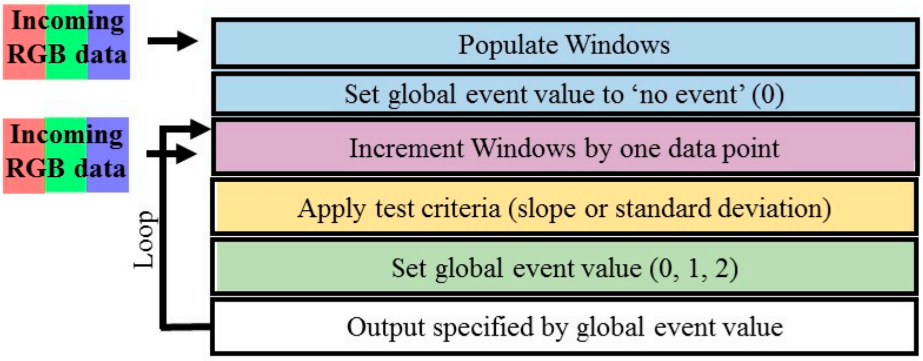

Global event value component common to both methods

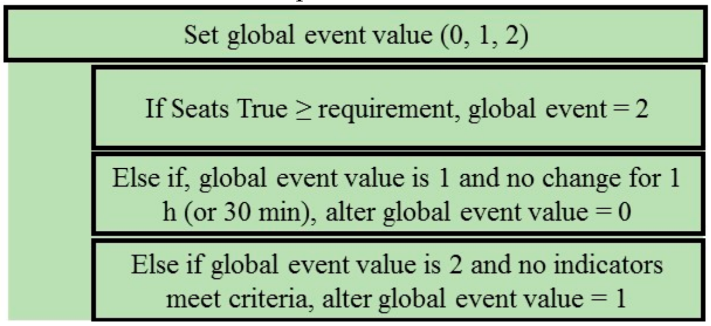

Standard deviation algorithm—populate windows

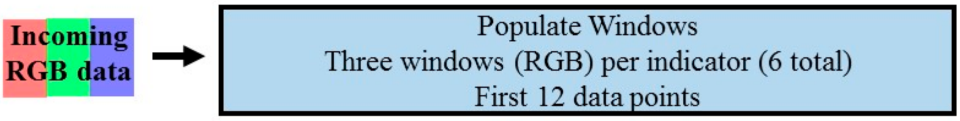

Standard deviation algorithm—window increment

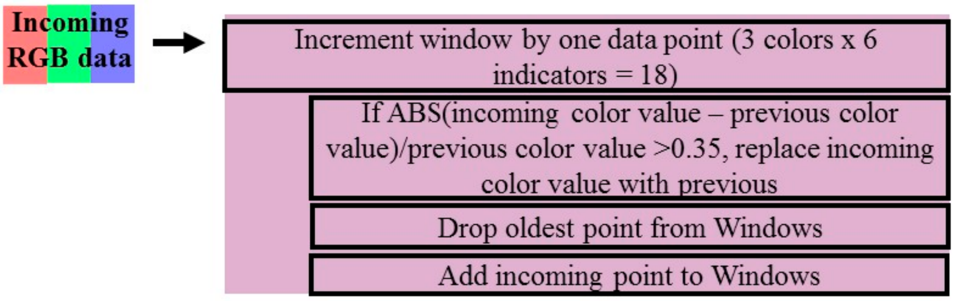

Standard deviation algorithm—test criteria

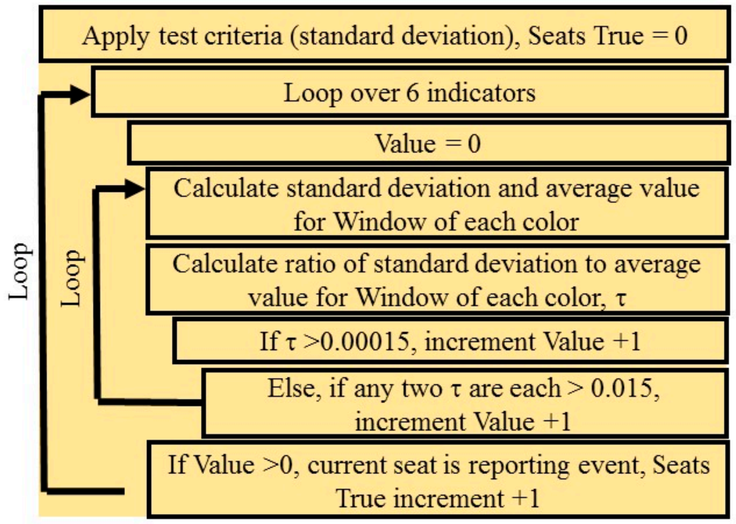

Slope algorithm—populate windows

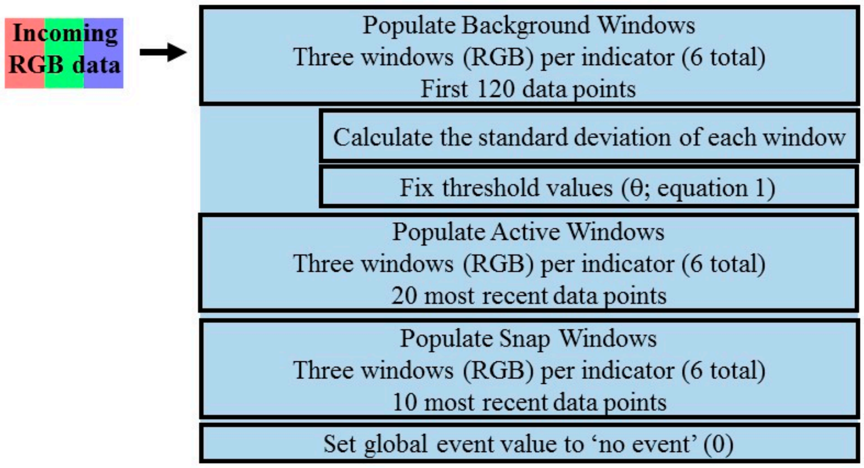

Slope algorithm—window increment

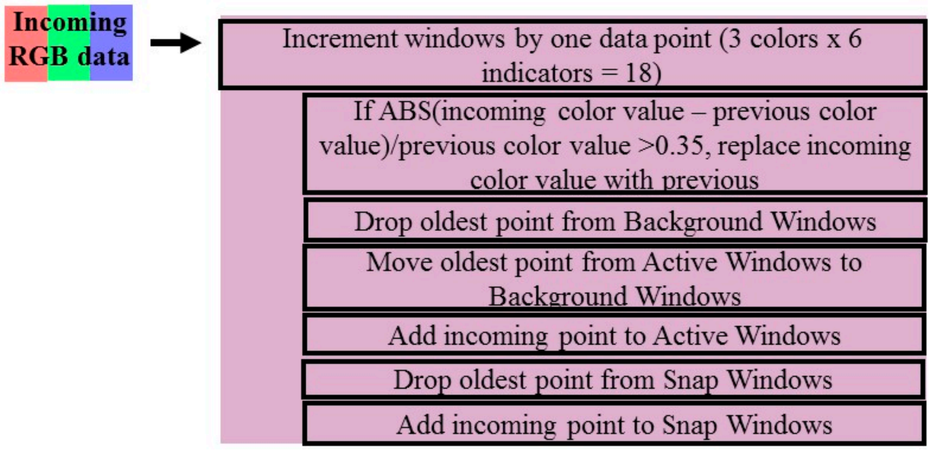

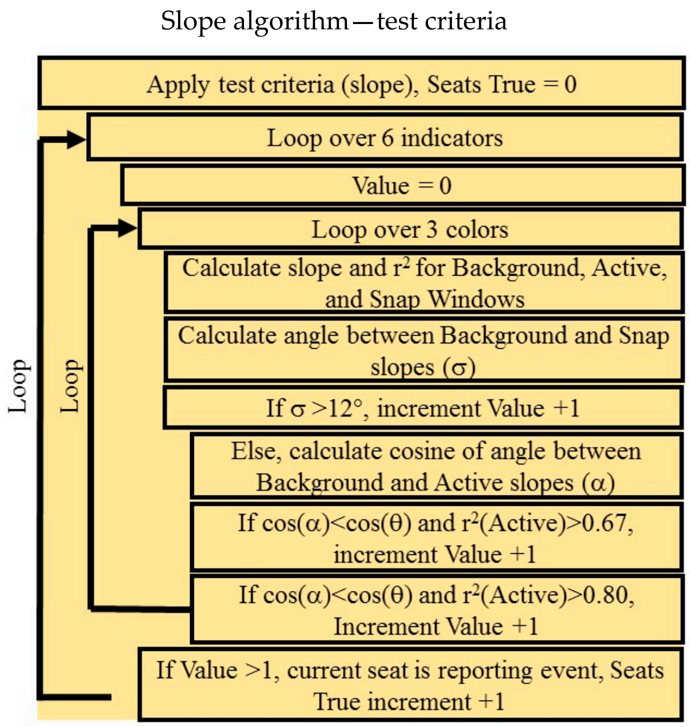
Slope algorithm—test criteria
Apply test criteria (slope), Seats True = 0
Loop over 6 indicators
Value = 0
Loop over 3 colors
Calculate slope and r2 for Background, Active, and Snap Windows
Calculate angle between Background and Snap slopes (σ)
If σ >12°, increment Value +1
Else, calculate cosine of angle between Background and Active slopes (α)
If cos(α)<cos(θ) and r2(Active)>0.67, increment Value +1
If cos(α)<cos(θ) and r2(Active)>0.80, Increment Value +1
If Value >1, current seat is reporting event, Seats True increment +1
Loop
Loop

Table S1. PT3 data set from Petri dish based exposures (5 s increment); total run time 175 h.

		Slope, 1 Seat (s1)			Slope, 2 Seats (s2)			Standard Deviation, 1s (s1std)			Standard Deviation, 2s (s2std)		
**Target (ppm)**	**Spiked**	**Detected Begin**	**Window End**	**Indicator #**	**Detected Begin**	**Window End**	**Indicator #**	**Detected Begin**	**Window End**	**Indicator #**	**Detected Begin**	**Window End**	**Indicator #**
Ethanol 8	1/28/2016 10:33	1/28/2016 10:42	1/28/2016 10:58	1;4;5;6	1/28/2016 10:42	1/28/2016 11:01	1;3;4;5;6	1/28/2016 10:44	1/28/2016 10:47	6			
Ethanol 8	1/28/2016 12:32	1/28/2016 12:31	1/28/2016 12:58	1;2;3;4;5;6	1/28/2016 12:31	1/28/2016 12:58	1;2;3;4;5;6	1/28/2016 12:31	1/28/2016 12:46	1;2;3;4;5;6	1/28/2016 12:31	1/28/2016 12:46	1;2;3;4;5;6
Ethanol 8	1/28/2016 14:07	1/28/2016 14:06	1/28/2016 14:37	1;2;3;4;5;6	1/28/2016 14:06	1/28/2016 14:40	1;2;3;4;5;6	1/28/2016 14:05	1/28/2016 14:17	1;2;3;4;5;6	1/28/2016 14:05	1/28/2016 14:06	1;2;3;4;5;6
Ethanol 16	1/29/2016 7:40	1/29/2016 7:38	1/29/2016 8:24	1;2;3;4;5;6	1/29/2016 7:39	1/29/2016 8:25	1;2;3;4;5;6	1/29/2016 7:38	1/29/2016 8:13	1;2;3;4;5;6	1/29/2016 7:38	1/29/2016 7:57	1;2;3;4;5;6
Ethanol 16	1/29/2016 9:40	1/29/2016 9:45	1/29/2016 10:33	1;2;3;4;5;6	1/29/2016 9:45	1/29/2016 10:33	1;2;3;4;5;6	1/29/2016 9:44	1/29/2016 10:22	1;2;3;4;5;6	1/29/2016 9:44	1/29/2016 10:15	1;2;3;4;5;6
Ethanol 16	1/29/2016 14:20	1/29/2016 13:46	1/29/2016 18:08	1;2;3;4;5;6	1/29/2016 14:20	1/29/2016 18:09	1;2;3;4;5;6	1/29/2016 14:19	1/29/2016 17:18	1;2;3;4;5;6	1/29/2016 14:20	1/29/2016 14:43	1;2;3;4;5;6
Ethanol 40	1/29/2016 15:58										1/29/2016 15:57	1/29/2016 17:02	1;2;3;4;5;6
Unknown		1/30/2016 13:41	1/30/2016 14:10	1;3;4									
Unknown		1/31/2016 14:02	1/31/2016 14:07	1									
Ethanol 40	2/1/2016 9:52	2/1/2016 9:52	2/1/2016 14:44	1;2;3;4;5;6	2/1/2016 9:53	2/1/2016 14:45	1;2;3;4;5;6	2/1/2016 9:51	2/1/2016 11:08	1;2;3;4;5;6	2/1/2016 9:52	2/1/2016 10:58	1;2;3;4;5;6
Ethanol 40	2/1/2016 12:16							2/1/2016 12:13	2/1/2016 13:41	1;2;3;4;5;6	2/1/2016 12:15	2/1/2016 13:21	1;2;3;4;5;6
Ethanol 61	2/1/2016 17:04	2/1/2016 17:04	2/1/2016 19:17	1;2;3;4;5;6	2/1/2016 17:04	2/1/2016 19:16	1;2;3;4;5;6	2/1/2016 17:03	2/1/2016 19:09	1;2;3;4;5;6	2/1/2016 17:03	2/1/2016 17:09	1;2;3;4;5;6
Unknown		2/1/2016 20:31	2/1/2016 21:47	1;3;5	2/1/2016 20:37	2/1/2016 21:30	1;2;3;4;5;6						
Unknown		2/2/2016 13:44	2/2/2016 14:07	1;2;3									
Ethanol 82	2/3/2016 9:22	2/3/2016 9:21	2/3/2016 10:56	1;2;3;4;5;6	2/3/2016 9:21	2/3/2016 10:56	1;2;3;4;5;6	2/3/2016 9:20	2/3/2016 10:49	1;2;3;4;5;6	2/3/2016 9:20	2/3/2016 10:22	1;2;3;4;5;6

Table S1. *Cont.*

		Slope, 1 Seat (s1)			Slope, 2 Seats (s2)			Standard Deviation, 1s (s1std)			Standard Deviation, 2s (s2std)		
Target (ppm)	Spiked	Detected Begin	Window End	Indicator #	Detected Begin	Window End	Indicator #	Detected Begin	Window End	Indicator #	Detected Begin	Window End	Indicator #
Unknown		2/3/2016 13:13	2/3/2016 13:24	1									
Ethanol 61	2/3/2016 14:29	2/3/2016 14:28	2/3/2016 16:02	1;2;3;4;5;6	2/3/2016 14:28	2/3/2016 15:34	1;2;3;4;5;6	2/3/2016 14:27	2/3/2016 15:56	1;2;3;4;5;6	2/3/2016 14:27	2/3/2016 15:26	1;2;3;4;5;6
Unknown		2/3/2016 18:45	2/3/2016 18:50	1									
Ethanol 61	2/4/2016 8:36	2/4/2016 8:35	2/4/2016 12:03	1;2;3;4;5;6	2/4/2016 8:35	2/4/2016 11:53	1;2;3;4;5;6	2/4/2016 8:34	2/4/2016 11:50	1;2;3;4;5;6	2/4/2016 8:34	2/4/2016 11:34	1;2;3;4;5;6
Ethanol 82	2/4/2016 10:15										2/4/2016 10:15	2/4/2016 11:15	1;2;3;4;5;6
Unknown		2/4/2016 13:12	2/4/2016 13:24	2				2/4/2016 13:14	2/4/2016 13:17	2			
Ethanol 82	2/5/2016 10:05	2/5/2016 10:12	2/5/2016 11:52	1;2;3;4;5;6	2/5/2016 10:12	2/5/2016 11:29	1;2;3;4;5;6				2/5/2016 10:51	2/5/2016 11:26	1;2;3;4;5;6
Unknown								2/5/2016 10:46	2/5/2016 11:42	1;2;3;4;5;6			

Table S2. PT3 data set from Petri dish based exposures (5 s increment); total run time 334 h.

		Slope, 1 Seat (s1)			Slope, 2 Seats (s2)			Standard Deviation, 1s (s1std)			Standard Deviation, 2s (s2std)		
Target (ppm)	Spiked	Detected Begin	Window End	Indicator #	Detected Begin	Window End	Indicator #	Detected Begin	Window End	Indicator #	Detected Begin	Window End	Indicator #
Ethanol 8	1/14/2016 12:33	1/14/2016 12:36	1/14/2016 12:55	1;2;3;4;5;6	1/14/2016 12:36	1/14/2016 13:02	1;2;3;4;5;6	1/14/2016 12:36	1/14/2016 12:43	1;2;3;4;6	1/14/2016 12:37	1/14/2016 12:43	1;2;3;4;6
Unknown		1/14/2016 14:00	1/14/2016 14:06	1									
Ethanol 8	1/14/2016 15:24	1/14/2016 15:23	1/14/2016 15:45	1;2;3;4;5;6	1/14/2016 15:23	1/14/2016 15:47	1;2;3;4;5;6	1/14/2016 15:23	1/14/2016 15:33	1;2;3;4;5;6	1/14/2016 15:23	1/14/2016 15:33	1;2;3;4;5;6
Ethanol 8	1/14/2016 16:28	1/14/2016 16:27	1/14/2016 16:56	1;2;3;4;5;6	1/14/2016 16:28	1/14/2016 16:57	1;2;3;4;5;6	1/14/2016 16:27	1/14/2016 16:40	1;2;3;4;5;6	1/14/2016 16:27	1/14/2016 16:40	1;2;3;4;5;6
Ethanol 16	1/14/2016 17:38	1/14/2016 17:38	1/14/2016 18:14	1;2;3;4;5;6	1/14/2016 17:39	1/14/2016 18:31	1;2;3;4;5;6	1/14/2016 17:38	1/14/2016 18:02	1;2;3;4;5;6	1/14/2016 17:38	1/14/2016 18:02	1;2;3;4;5;6

Table S2. *Cont.*

		Slope, 1 Seat (s1)			Slope, 2 Seats (s2)			Standard Deviation, 1s (s1std)			Standard Deviation, 2s (s2std)		
**Target (ppm)**	**Spiked**	**Detected Begin**	**Window End**	**Indicator #**	**Detected Begin**	**Window End**	**Indicator #**	**Detected Begin**	**Window End**	**Indicator #**	**Detected Begin**	**Window End**	**Indicator #**
Unknown		1/15/2016 9:40	1/15/2016 9:40										
Ethanol 16	1/15/2016 9:49	1/15/2016 9:55	1/15/2016 10:38	1;2;3;4;5;6	1/15/2016 9:55	1/15/2016 10:50	1;2;3;4;5;6	1/15/2016 9:55	1/15/2016 10:19	1;2;3;4;5;6	1/15/2016 9:55	1/15/2016 10:19	1;2;3;4;5;6
Ethanol 16	1/15/2016 12:57	1/15/2016 12:57	1/15/2016 13:47	1;2;3;4;5;6	1/15/2016 12:57	1/15/2016 13:48	1;2;3;4;5;6	1/15/2016 12:56	1/15/2016 13:35	1;2;3;4;5;6	1/15/2016 12:56	1/15/2016 13:35	1;2;3;4;5;6
Ethanol 40	1/15/2016 15:42	1/15/2016 15:42	1/15/2016 18:05	1;2;3;4;5;6	1/15/2016 15:42	1/15/2016 16:49	1;2;3;4;5;6	1/15/2016 15:42	1/15/2016 16:35	1;2;3;4;5;6	1/15/2016 15:42	1/15/2016 16:29	1;2;3;4;5;6
Unknown		1/18/2016 13:53	1/18/2016 14:12	1;2;3				1/15/2016 17:35	1/15/2016 17:35	1			
Unknown								1/16/2016 13:57	1/16/2016 14:07	1;2			
Unknown								1/18/2016 13:56	1/18/2016 14:10	1;2			
Ethanol 40	1/19/2016 10:53	1/19/2016 10:53	1/19/2016 13:10	1;2;3;4;5;6	1/19/2016 10:53	1/19/2016 13:11	1;2;3;4;5;6	1/19/2016 10:52	1/19/2016 12:57	1;2;3;4;5;6	1/19/2016 10:53	1/19/2016 10:59	1;2;3;4;5;6
Unknown											1/19/2016 12:03	1/19/2016 12:10	1;3;5;6
Ethanol 40	1/19/2016 14:33	1/19/2016 13:45	1/19/2016 16:44	1;2;3;4;5;6	1/19/2016 14:32	1/19/2016 16:21	1;2;3;4;5;6	1/19/2016 14:32	1/19/2016 14:38	1;2;3;4;5;6	1/19/2016 14:32	1/19/2016 14:37	1;2;3;4;5;6
Unknown								1/19/2016 15:13	1/19/2016 16:17	1;2;3;4;5;6	1/19/2016 15:20	1/19/2016 15:31	1;3;4;5
Ethanol 61	1/19/2016 17:37	1/19/2016 17:15	1/19/2016 19:59	1;2;3;4;5;6	1/19/2016 17:17	1/19/2016 19:47	1;2;3;4;5;6	1/19/2016 17:39	1/19/2016 18:17	1;2;3;4;5;6	1/19/2016 17:39	1/19/2016 17:45	1;2;3;4;5;6
Unknown		1/19/2016 22:44	1/19/2016 22:46	4	1/19/2016 21:05	1/19/2016 21:39	2;3;4;6	1/19/2016 19:15	1/19/2016 19:44	2;3;5;6	1/19/2016 19:16	1/19/2016 19:18	2;3;5
								1/19/2016 20:54	1/19/2016 20:59	4			
Ethanol 61	1/21/2016 9:47	1/21/2016 9:46	1/21/2016 11:55	1;2;3;4;5;6	1/21/2016 9:46	1/21/2016 11:24	1;2;3;4;5;6	1/21/2016 9:46	1/21/2016 9:52	1;2;3;4;5;6	1/21/2016 9:46	1/21/2016 9:51	1;2;3;4;5;6

Table S2. *Cont.*

		Slope, 1 Seat (s1)			Slope, 2 Seats (s2)			Standard Deviation, 1s (s1std)			Standard Deviation, 2s (s2std)		
**Target (ppm)**	**Spiked**	**Detected Begin**	**Window End**	**Indicator #**	**Detected Begin**	**Window End**	**Indicator #**	**Detected Begin**	**Window End**	**Indicator #**	**Detected Begin**	**Window End**	**Indicator #**
Unknown								1/21/2016 10:23	1/21/2016 11:21	1;2;3;4;5;6			
Ethanol 61	1/21/2016 13:12	1/21/2016 12:42	1/21/2016 20:04	1;2;3;4;5;6	1/21/2016 13:11	1/21/2016 19:16	1;2;3;4;5;6	1/21/2016 13:10	1/21/2016 14:53	1;2;3;4;5;6	1/21/2016 13:10	1/21/2016 13:15	1;2;3;4;5;6
Unknown								1/21/2016 15:33	1/21/2016 15:51	1;2;3;4;5;6	1/21/2016 15:46	1/21/2016 15:51	1;2;3;4;5;6
Ethanol 82	1/21/2016 17:29							1/21/2016 16:40	1/21/2016 18:13	1;2;3;4;5;6	1/21/2016 17:28	1/21/2016 17:33	1;2;3;4;5;6
Unknown		1/21/2016 20:44	1/21/2016 20:44	2				1/21/2016 18:45	1/21/2016 19:29	2;3;4;5;6			
Unknown		1/21/2016 23:51	1/22/2016 0:21	5									
Ethanol 82	1/22/2016 7:34	1/22/2016 7:34	1/22/2016 9:22	2	1/22/2016 7:35	1/22/2016 9:06	1;2;3;4;5;6	1/22/2016 7:34	1/22/2016 9:16	1;2;3;4;5;6	1/22/2016 7:34	1/22/2016 7:39	1;2;3;4;5;6
Unknown											1/22/2016 8:22	1/22/2016 8:42	1;3;4;5;6
Ethanol 82	1/22/2016 11:18	1/22/2016 10:34	1/22/2016 13:14	4	1/22/2016 10:58	1/22/2016 12:49	1;2;3;4;5;6	1/22/2016 11:17	1/22/2016 12:36	1;2;3;4;5;6	1/22/2016 11:17	1/22/2016 11:48	1;2;3;4;5;6
Unknown		1/22/2016 16:22	1/22/2016 16:29	1	1/22/2016 14:03	1/22/2016 14:04	1;2	1/22/2016 13:07	1/22/2016 13:08	5			
Unknown		1/24/2016 13:36	1/24/2016 14:13	4	1/22/2016 16:22	1/22/2016 16:29	1;4	1/24/2016 14:03	1/24/2016 14:06	1			
Unknown		1/25/2016 14:12	1/25/2016 14:12	1	1/24/2016 13:52	1/24/2016 13:53	1;2;3	1/27/2016 14:02	1/27/2016 14:05	1			
Unknown		1/27/2016 13:39	1/27/2016 14:07	4									

Table S3. PT3 data set from enclosure based exposures (5 s increment); total run time 1004 h.

		**Slope, 1 Seat (s1)**			**Slope, 2 Seats (s2)**			**Standard Deviation, 1s (s1std)**			**Standard Deviation, 2s (s2std)**		
**Target (ppm)**	**Spiked**	**Detected Begin**	**Window End**	**Indicator #**	**Detected Begin**	**Window End**	**Indicator #**	**Detected Begin**	**Window End**	**Indicator #**	**Detected Begin**	**Window End**	**Indicator #**
Ethanol 0.16	12/4/2015 9:30	12/4/2015 9:30	12/4/2015 9:32	4;5;6	12/4/2015 9:29	12/4/2015 9:32	1;2;4;5;6						
Ethanol 0.16	12/4/2015 12:42	12/4/2015 12:42	12/4/2015 12:45	1;2;4;5;6	12/4/2015 12:42	12/4/2015 12:45	1;2;4;5;6						
Unknown		12/4/2015 14:00	12/4/2015 14:05	1	12/4/2015 14:01	12/4/2015 14:05	1;5;6						
Ethanol 0.16	12/4/2015 16:10	12/4/2015 16:10	12/4/2015 16:13	1;2;4;5;6	12/4/2015 16:09	12/4/2015 16:14	1;2;3;4;5;6						
Ethanol 0.32	12/7/2015 13:22	12/7/2015 13:22	12/7/2015 13:29	1;2;4;6	12/7/2015 13:22	12/7/2015 13:29	1;2;4;6						
Ethanol 0.32	12/7/2015 15:24	12/7/2015 15:05	12/7/2015 15:12	1;2;4;5;6	12/7/2015 15:05	12/7/2015 15:12	1;2;4;5;6						
Ethanol 0.32	12/7/2015 17:40	12/7/2015 17:40	12/7/2015 17:46	1;2;4;6	12/7/2015 17:40	12/7/2015 17:47	1;2;4;6						
Ethanol 0.53	12/9/2015 8:02	12/9/2015 8:07	12/9/2015 8:19	1;2;4;5;6	12/9/2015 8:02	12/9/2015 8:21	1;2;4;5;6						
Ethanol 0.53	12/9/2015 11:51	12/9/2015 11:52	12/9/2015 12:11	1;2;3;4;5;6	12/9/2015 11:52	12/9/2015 12:16	1;2;3;4;5;6						
Ethanol 0.53	12/10/2015 12:00	12/10/2015 12:02	12/10/2015 12:16	1;2;4;5;6	12/10/2015 12:01	12/10/2015 12:17	1;2;3;4;5;6						
Ethanol 1.06	12/11/2015 10:50	12/11/2015 10:48	12/11/2015 11:17	1;2;3;4;5;6	12/11/2015 10:47	12/11/2015 11:19	1;2;3;4;5;6	12/11/2015 10:57	12/11/2015 11:06	2;4;6	12/11/2015 10:57	12/11/2015 11:06	2;4;6
Unknown								12/11/2015 14:07	12/11/2015 14:19	1;2;4;6	12/11/2015 14:07	12/11/2015 14:19	1;2;4;6
Unknown								12/11/2015 14:47	12/11/2015 14:47	1;4	12/11/2015 14:47	12/11/2015 14:47	1;4
Ethanol 1.06	12/11/2015 15:26	12/11/2015 14:46	12/11/2015 16:03	1;2;3;4;5;6	12/11/2015 14:48	12/11/2015 15:57	1;2;3;4;5;6	12/11/2015 15:46	12/11/2015 15:49	4;5;6	12/11/2015 15:46	12/11/2015 15:49	4;5;6
Ethanol 0.32	12/21/2015 15:19	12/21/2015 15:21	12/21/2015 15:27	1;2;4;5	12/21/2015 15:21	12/21/2015 15:28	1;2;3;4;5						

Table S3. *Cont.*

		Slope, 1 Seat (s1)			Slope, 2 Seats (s2)			Standard Deviation, 1s (s1std)			Standard Deviation, 2s (s2std)		
Target (ppm)	Spiked	Detected Begin	Window End	Indicator #	Detected Begin	Window End	Indicator #	Detected Begin	Window End	Indicator #	Detected Begin	Window End	Indicator #
Ethanol 0.32	12/21/2015 17:53	12/21/201 5 17:54	12/21/201 5 18:00	1;2;4;6	12/21/201 5 17:54	12/21/201 5 18:08	1;2;4;5;6						
Ethanol 0.32	12/23/2015 9:52	12/23/201 5 10:00	12/23/201 5 10:06	1;2;4;6	12/23/201 5 9:59	12/23/201 5 10:07	1;2;4;5;6						
Ethanol 0.53	12/23/2015 12:19	12/23/201 5 12:22	12/23/201 5 12:36	1;2;4;5;6	12/23/201 5 12:22	12/23/201 5 12:37	1;2;3;4;5;6						
Ethanol 0.53	12/23/2015 16:20	12/23/201 5 16:25	12/23/201 5 16:42	1;2;4;6	12/23/201 5 16:25	12/23/201 5 16:43	1;2;4;5;6						
Unknown		1/4/2016 15:50	1/4/2016 15:50	4	1/9/2016 22:08	1/9/2016 22:08	2;4	1/11/2016 16:08	1/11/2016 16:08	1;3;6	1/11/2016 16:08	1/11/2016 16:08	1;3;6
Ethanol 0.53	1/11/2016 18:13	1/11/2016 18:13	1/11/2016 18:18	1;4;5;6	1/11/2016 18:13	1/11/2016 18:22	1;2;4;5;6	1/11/2016 18:12	1/11/2016 18:13	4;6	1/11/2016 18:12	1/11/2016 18:13	4;6
Ethanol 1.06	1/12/2016 9:26	1/12/2016 9:28	1/12/2016 9:53	3;4;5;6	1/12/2016 9:27	1/12/2016 9:54	1;2;3;4;5;6						
Ethanol 1.06	1/12/2016 12:35	1/12/2016 12:34	1/12/2016 12:53	1;2;3;4;5;6	1/12/2016 12:34	1/12/2016 12:56	1;2;3;4;5;6						
Ethanol 1.06	1/12/2016 17:26	1/12/2016 17:25	1/12/2016 17:40	1;2;3;4;5;6	1/12/2016 17:25	1/12/2016 17:41	1;2;3;4;5;6						
Ethanol 1.58	1/13/2016 10:52	1/13/2016 10:52	1/13/2016 11:26	1;2;3;4;5;6	1/13/2016 10:51	1/13/2016 11:26	1;2;3;4;5;6	1/13/2016 11:12	1/13/2016 11:13	5;6	1/13/2016 11:12	1/13/2016 11:13	5;6
Ethanol 1.58	1/13/2016 14:02	1/13/2016 14:02	1/13/2016 15:27	1;2;3;4;5;6	1/13/2016 14:02	1/13/2016 14:29	1;2;3;4;5;6						
Ethanol 1.58	1/13/2016 17:03	1/13/2016 17:03	1/13/2016 17:24	1;2;3;4;5;6	1/13/2016 17:03	1/13/2016 17:29	1;2;3;4;5;6						

Table S4. Analysis of PT3 data sets. Specificity is calculated as the total true negatives divided by the sum of the true negatives and the false positives. Sensitivity is calculated as the total true positives divided by the sum of the true positives and the false negatives.

Device	Integration (ms)	Interval (s)	Experiment Type	Threshold (#Seats) †	Algorithm Type	True Positives	False Positives	False Negatives	True Negatives	Specificity	Sensitivity
PT3	100	5	Petri	1	St. Dev. (s1std)	156	45	24	491	0.916	0.87
PT3	100	5	Petri	2	St. Dev. (s2std)	167	22	13	503	0.958	0.93
PT3	100	5	Petri	1	Slope (s1)	154	30	26	524	0.946	0.86
PT3	100	5	Petri	2	Slope (s2)	155	17	25	476	0.966	0.86
PT3	100	5	Enclosure	1	St. Dev. (s1std)	10	9	128	982	0.991	0.07
PT3	100	5	Enclosure	2	St. Dev. (s2std)	10	9	128	982	0.991	0.07
PT3	100	30	Enclosure	1	St. Dev. (s1std)	75	13	63	923	0.986	0.54
PT3	100	30	Enclosure	2	St. Dev. (s2std)	52	7	86	896	0.992	0.38
PT3	100	5	Enclosure	1	Slope (s1)	113	2	25	997	0.998	0.82
PT3	100	5	Enclosure	2	Slope (s2)	127	5	11	995	0.995	0.92
PT3	100	30	Enclosure	1	Slope (s1)	113	6	25	967	0.994	0.82
PT3	100	30	Enclosure	2	Slope (s2)	128	8	10	978	0.992	0.93

† Data sets from the Petri dish based experiments included concentrations from 8 to 82 ppm; concentrations from the enclosure ranged from 0.16 to 1.58 ppm.

Table S5. PT5 data set from enclosure based exposures (5 s increment; 100 ms integration); total run time 243 h. Results presented for one device of four total.

		Slope, 1 Seat (s1)			Slope, 2 Seats (s2)			Standard Deviation, 1s (s1std)			Standard Deviation, 2s (s2std)		
Target (ppm)	Spiked	Detected Begin	Window End	Indicator #	Detected Begin	Window End	Indicator #	Detected Begin	Window End	Indicator #	Detected Begin	Window End	Indicator #
Unknown		12/4/2015 5:23	12/4/2015 5:23	6									
Unknown		12/4/2015 6:26	12/4/2015 6:27	6	12/4/2015 6:30	12/4/2015 6:32	2;3;6						
Ethanol 0.16	12/4/2015 9:30	12/4/2015 9:27	12/4/2015 10:51	1;4;5;6	12/4/2015 9:23	12/4/2015 9:24	4;6		**No Reported Events**			**No Reported Events**	
Ethanol 0.16	12/4/2015 12:42	12/4/2015 12:41	12/4/2015 14:23	1;4;6									
Ethanol 0.16	12/4/2015 16:10	12/4/2015 16:11	12/4/2015 16:30	1;4;5									

Table S5. *Cont.*

		Slope, 1 Seat (s1)			Slope, 2 Seats (s2)			Standard Deviation, 1s (s1std)			Standard Deviation, 2s (s2std)		
**Target (ppm)**	**Spiked**	**Detected Begin**	**Window End**	**Indicator #**	**Detected Begin**	**Window End**	**Indicator #**	**Detected Begin**	**Window End**	**Indicator #**	**Detected Begin**	**Window End**	**Indicator #**
Unknown		12/5/2015 0:09	12/5/2015 0:09	3									
Unknown		12/5/2015 1:26	12/5/2015 1:26	3									
Unknown		12/5/2015 4:34	12/5/2015 4:35	3									
Ethanol 1.06	12/11/2015 15:26	12/11/2015 14:39	12/11/2015 16:28	1;2;4;5;6									
Unknown		12/12/2015 8:25	12/12/2015 8:25	4									
Unknown		12/12/2015 9:55	12/12/2015 9:55	2	12/11/2015 19:06	12/11/2015 20:01	2;3;6						
Unknown		12/12/2015 13:19	12/12/2015 15:02	1;2;3;4	12/12/2015 14:05	12/12/2015 14:06	2;3;6						
Ethanol 0.32	12/21/2015 15:19	12/21/2015 13:34	12/21/2015 16:49	1;3;4;5									
Ethanol 0.32	12/21/2015 17:53	12/21/2015 18:04	12/21/2015 19:53	1;2;3									
Ethanol 0.32	12/23/2015 9:52	12/23/2015 10:14	12/23/2015 10:19	1									
Ethanol 0.53	12/23/2015 12:19	12/23/2015 12:19	12/23/2015 14:26	1;2;4;6									
Ethanol 0.53	12/23/2015 16:20	12/23/2015 16:42	12/23/2015 17:49	1									
Unknown		12/23/2015 22:20	12/24/2015 1:02	1;2;4;6									
Ethanol 0.53	1/11/2016 18:13	1/11/2016 16:13	1/11/2016 19:56	1;2;3;4									

Table S5. *Cont.*

		Slope, 1 Seat (s1)			Slope, 2 Seats (s2)			Standard Deviation, 1s (s1std)			Standard Deviation, 2s (s2std)		
**Target (ppm)**	**Spiked**	**Detected Begin**	**Window End**	**Indicator #**	**Detected Begin**	**Window End**	**Indicator #**	**Detected Begin**	**Window End**	**Indicator #**	**Detected Begin**	**Window End**	**Indicator #**
Unknown		1/12/2016 2:09	1/12/2016 2:12	3									
Unknown		1/12/2016 3:39	1/12/2016 3:51	3									
Ethanol 1.06	1/12/2016 9:26	1/12/2016 9:28	1/12/2016 15:50	1;2;3;4;5	1/12/2016 12:26	1/12/2016 12:49	2;6						
Ethanol 1.06	1/12/2016 12:35	1/12/2016 18:02	1/12/2016 19:30	1;5									
Unknown		1/13/2016 2:40	1/13/2016 2:42	3	1/12/2016 19:02	1/12/2016 19:20	1;6						
Unknown		1/13/2016 7:31	1/13/2016 7:31	3	1/13/2016 8:54	1/13/2016 8:54	2;3;6						
Ethanol 1.58	1/13/2016 10:52	1/13/2016 8:54	1/13/2016 11:28	1;2;4;5;6	1/13/2016 11:01	1/13/2016 11:16	1;2;3;4;5;6						
Ethanol 1.58	1/13/2016 14:02	1/13/2016 12:33	1/13/2016 20:26	1;2;4;5;6	1/13/2016 14:11	1/13/2016 14:16	1;2;3;6						
Ethanol 1.58	1/13/2016 17:03				1/13/2016 17:13	1/13/2016 17:16	2;3;6						
Unknown		1/13/2016 21:33	1/13/2016 21:33	1									

Table S6. Analysis of PT5 data sets. Specificity is calculated as the total true negatives divided by the sum of the true negatives and the false positives. Sensitivity is calculated as the total true positives divided by the sum of the true positives and the false negatives.

Device	Integration (ms)	Interval (s)	Experiment Type	Threshold † (#Seats)	Algorithm Type	True Positives	False Positives	False Negatives	True Negatives	Specificity	Sensitivity
PT5	100	5	Enclosure	1	St. Dev. (s1std)	0	1	384	435	0.998	0.00
PT5	100	30	Enclosure	1	St. Dev. (s1std)	8	5	376	487	0.990	0.02
PT5	100	5	Enclosure	1	Slope (s1)	192	85	192	753	0.899	0.50
PT5	100	5	Enclosure	2	Slope (s2)	126	68	258	710	0.913	0.33
PT5	100	30	Enclosure	1	Slope (s1)	85	50	281	754	0.938	0.23
PT5	100	30	Enclosure	2	Slope (s2)	184	11	200	552	0.980	0.48
PT5	200	30	Enclosure	1	Slope (s1)	216	0	144	1072	1.000	0.60
PT5	200	30	Enclosure	2	Slope (s2)	259	9	101	1086	0.992	0.72
PT5	400	30	Enclosure	1	Slope (s1)	295	0	65	1234	1.000	0.82
PT5	400	30	Enclosure	2	Slope (s2)	314	0	46	1232	1.000	0.87
PT5	400	30	Enclosure	3	Slope (s3)	308	0	46	1254	1.000	0.87
PT5	400	30	Enclosure	2 (t2)	Slope (s2t2)	255	0	105	1262	1.000	0.71
PT5	400	30	Enclosure	2 (t3)	Slope (s2t3)	205	0	155	1269	1.000	0.57
PT5	500	30	Enclosure	1	Slope (s1)	271	1	65	1071	0.999	0.81
PT5	500	30	Enclosure	2	Slope (s2)	291	4	45	1079	0.996	0.87
PT5	500	30	Enclosure	3	Slope (s3)	285	0	51	1071	1.000	0.85
PT5	500	30	Enclosure	1 (t2)	Slope (s1t2)	224	0	112	1071	1.000	0.67
PT5	500	30	Enclosure	1 (t3)	Slope (s1t3)	176	0	160	1071	1.000	0.52
PT5	500	30	Enclosure	1 (50 ppb)	Slope (s1)	274	1	134	1076	0.999	0.67
PT5	500	30	Enclosure	2 (50 ppb)	Slope (s2)	294	4	114	1079	0.996	0.72
PT5	500	30	Enclosure	3 (50 ppb)	Slope (s3)	285	0	123	1071	1.000	0.70
PT5	500	30	Enclosure	1 (t2, 50 ppb)	Slope (s1t2)	227	0	181	1071	1.000	0.56
PT5	500	30	Enclosure	1 (t3, 50 ppb)	Slope (s1t3)	176	0	232	1071	1.000	0.43

† Threshold values for the number of responsive seats, the change in slope, and the concentration can be adjusted. Unless indicated, the data set uses 160 ppb as the intended detection limit. Unless indicated, the threshold angle is 0.45°; t2 = 1.45°, t3 = 2.45°.

Table S7. Sensor performance for varied integration time.

Integration (ms)	Red		Green		Blue		Overall	
	Average	Standard Deviation	Average	Standard Deviation	Average	Standard Deviation	Average	%
PT5 100	113	4.6	27	1.0	28	1.3	56	4.4
PT5 200	231	2.5	57	0.60	63	0.71	117	2.8
PT5 300 *	657	11	174	3.2	222	4.2	351	1.8
PT5 400	456	10	110	1.7	117	1.7	227	1.7
PT5 500	713	25	275	8.2	158	2.3	326	0.73
PT3	569	2.6	437	1.6	391	1.5	466	0.42

* Total signal impacted by the use of mixed indicator materials; all other values based on AgN_4TPP only.

Table S8. PT5 data set from enclosure based exposures (30 s increment; 500 ms integration); total run time 510 h.

		Slope, 1 Seat			Slope, 2 Seats			Slope, 3 Seats			Slope, 1 Seat, 1.45°		
Target (ppm)	Spiked	Detected Begin	Window End	Indicator #	Detected Begin	Window End	Indicator #	Detected Begin	Window End	Indicator #	Detected Begin	Window End	Indicator #
Ethanol 0.05	2/9/2016 12:55	2/9/2016 12:58	2/9/2016 13:01	6							2/9/2016 12:58	2/9/2016 13:01	6
Ethanol 0.05	2/9/2016 15:45	2/9/2016 15:48	2/9/2016 15:52	6							2/9/2016 15:48	2/9/2016 15:51	6
Ethanol 0.05	2/9/2016 17:45	2/9/2016 17:48	2/9/2016 18:24	6							2/9/2016 17:48	2/9/2016 17:51	6
Ethanol 0.16	2/11/2016 10:37	2/11/2016 10:41	2/11/2016 11:34	1;6	2/11/2016 10:42	2/11/2016 10:53	1;2;6	2/11/2016 10:43	2/11/2016 10:53	1;2;6	2/11/2016 10:41	2/11/2016 10:50	6
Ethanol 0.16	2/11/2016 13:37	2/11/2016 13:40	2/11/2016 14:35	1;2;6	2/11/2016 13:41	2/11/2016 13:50	1;2;6	2/11/2016 13:43	2/11/2016 13:50	1;2;6	2/11/2016 13:41	2/11/2016 13:48	6
Ethanol 0.16	2/11/2016 17:38	2/11/2016 17:41	2/11/2016 18:33	1;2;6	2/11/2016 17:42	2/11/2016 17:49	1;2;6	2/11/2016 17:44	2/11/2016 17:49	1;2;6	2/11/2016 17:41	2/11/2016 17:48	6
Ethanol 0.32	2/12/2016 10:03	2/12/2016 10:07	2/12/2016 11:07	1;2;3;4;5;6	2/12/2016 10:09	2/12/2016 11:08	1;2;3;4;5;6	2/12/2016 10:10	2/12/2016 10:21	1;2;3;4;5;6	2/12/2016 10:07	2/12/2016 10:58	1;2;4;6
Ethanol 0.32	2/12/2016 14:42	2/12/2016 14:45	2/12/2016 15:44	1;2;3;4;5;6	2/12/2016 14:45	2/12/2016 14:56	1;2;3;4;5;6	2/12/2016 14:45	2/12/2016 14:56	1;2;3;4;5;6	2/12/2016 14:45	2/12/2016 15:37	1;2;6

Table S8. *Cont.*

		Slope, 1 Seat			Slope, 2 Seats			Slope, 3 Seats			Slope, 1 Seat, 1.45°		
**Target (ppm)**	**Spiked**	**Detected Begin**	**Window End**	**Indicator #**	**Detected Begin**	**Window End**	**Indicator #**	**Detected Begin**	**Window End**	**Indicator #**	**Detected Begin**	**Window End**	**Indicator #**
Ethanol 0.32	2/16/2016 15:11	2/16/2016 15:15	2/16/2016 16:15	1;2;3;4;5;6	2/16/2016 15:14	2/16/2016 15:57	1;2;3;4;5;6	2/16/2016 15:15	2/16/2016 15:30	1;2;3;4;5;6	2/16/2016 15:17	2/16/2016 15:52	1;2;3;6
Ethanol 0.53	2/17/2016 9:27	2/17/2016 9:31	2/17/2016 10:37	1;2;3;4;5;6	2/17/2016 9:33	2/17/2016 10:37	1;2;3;4;5;6	2/17/2016 9:34	2/17/2016 10:37	1;2;3;4;5;6	2/17/2016 9:32	2/17/2016 10:31	1;2;3;4;5;6
Ethanol 0.53	2/17/2016 12:49	2/17/2016 12:53	2/17/2016 13:56	1;2;3;4;5;6	2/17/2016 12:52	2/17/2016 13:56	1;2;3;4;5;6	2/17/2016 12:54	2/17/2016 13:56	1;2;3;4;5;6	2/17/2016 12:53	2/17/2016 13:49	1;2;3;4;5;6
Ethanol 0.53	2/17/2016 15:27	2/17/2016 15:31	2/17/2016 16:33	1;2;3;4;5;6	2/17/2016 15:31	2/17/2016 16:34	1;2;3;4;5;6	2/17/2016 15:32	2/17/2016 16:34	1;2;3;4;5;6	2/17/2016 15:31	2/17/2016 16:27	1;2;3;4;5;6
Ethanol 1.06	2/18/2016 10:00	2/18/2016 10:07	2/18/2016 12:01	1;2;3;4;5;6	2/18/2016 10:10	2/18/2016 12:02	1;2;3;4;5;6	2/18/2016 10:10	2/18/2016 11:51	1;2;3;4;5;6	2/18/2016 10:07	2/18/2016 11:20	1;2;3;4;5;6
Ethanol 1.06	2/18/2016 15:00	2/18/2016 15:05	2/18/2016 16:28	1;2;3;4;5;6	2/18/2016 15:06	2/18/2016 16:29	1;2;3;4;5;6	2/18/2016 15:06	2/18/2016 16:29	1;2;3;4;5;6	2/18/2016 15:06	2/18/2016 16:16	1;2;3;4;5;6
Ethanol 1.58	2/23/2016 13:10	2/23/2016 13:14	2/23/2016 15:17	6	2/23/2016 13:16	2/23/2016 14:58	1;2;3;4;5;6	2/23/2016 13:16	2/23/2016 14:58	1;2;3;4;5;6	2/23/2016 13:14	2/23/2016 14:47	1;2;3;4;5;6
Ethanol 1.58	2/24/2016 13:20	2/24/2016 13:23	2/24/2016 15:30	6	2/24/2016 13:24	2/24/2016 15:26	1;2;3;4;5;6	2/24/2016 13:24	2/24/2016 15:04	1;2;3;4;5;6	2/24/2016 13:23	2/24/2016 14:47	1;2;3;4;5;6
Ethanol 1.58	2/25/2016 13:13	2/25/2016 13:16	2/25/2016 15:25	6	2/25/2016 13:18	2/25/2016 14:55	1;2;3;4;5;6	2/25/2016 13:18	2/25/2016 14:55	1;2;3;4;5;6	2/25/2016 13:16	2/25/2016 14:42	1;2;3;4;5;6
Unknown		2/28/2016 8:31	2/28/2016 8:33	3									
